# Friction Stir Welding of 5754 Aluminum Alloy with Cover Sheet

**DOI:** 10.3390/ma12111765

**Published:** 2019-05-31

**Authors:** Daxin Ren, Fanyu Zeng, Yi Liu, Liming Liu, Zhubin He

**Affiliations:** 1 School of Automotive Engineering, Dalian University of Technology, Dalian 116024, China; rendx@dlut.edu.cn (D.R.); s853619@mail.dlut.edu.cn (F.Z.); 15265284530@163.com (Y.L.); 2 School of Materials Science and Engineering, Dalian University of Technology, Dalian 116024, China; 3 School of Mechanical Engineering, Dalian University of Technology, Dalian 116024, China; hezb@dlut.edu.cn

**Keywords:** friction stir welding with cover sheet, weld reinforcement, mechanical properties, microstructure

## Abstract

Friction stir welding can realize high-strength aluminum alloy joints. In this study, friction stir welding with cover sheet (CFSW) is proposed to solve the thinning caused by the tool shoulder and reduce the heat-affected zone. The microstructures and mechanical properties of CFWS were also studied. After the cover sheet was added, a reinforcement was formed on the weld surface, which compensated the thinning caused by the friction of the tool shoulder. As the cover absorbed heat from the shoulder, the width of the heat-affected zone of the welded sheet became smaller than the diameter of the shoulder. Without milling the cover sheet, the tensile strength of the 5754 aluminum alloy joint reached 94% of that of the base metal. The fracture position was the heat-affected zone of the forward-side weld joint. After the cover sheet was added, the stress concentration shifted from the thinning area of traditional friction stir welding to the outside of the welding seam.

## 1. Introduction

Aluminum alloys have been widely used in shipbuilding, automotive, and aerospace industries due to their excellent mechanical properties (e.g., rigidity and strength–weight ratio) [1]. At present, friction stir welding is widely used in the welding of aluminum alloys due to its excellent mechanical properties [2,3,4].

During friction stir welding, the tool pin is inserted into the welded sheet. Heat is generated by the friction between the shoulder and the upper surface of the sheet and the stirring action of the tool pin in the workpiece. The material flows with the tool pin and fills the cavity, thereby forming a compact weld seam structure [5]. Welding strength is primarily affected by five parameters, namely, rotating speed, welding speed, plunge depth, inclination, and tool shape [6,7,8,9,10]. To form a compact weld seam, a suitable plunge depth must be selected for the tool. However, this condition presses the shoulder into the weld seam area and causes inevitable thinning of the weld seam of the sheet, thereby reducing the mechanical properties. Moreover, failure occurs in the subsequent plastic forming process [11]. When welding 6061 and other aluminum alloys, the heat-affected zone on both sides of the weld seam, which is the primary site affecting the mechanical properties, softens [12].

At present, to solve the thinning of the weld seam, special tools or welding additive equipment are often used, or the thickness of the partial sheet for welding is increased in the rolling process [13]. In other melt welding methods (e.g., laser or arc welding), reinforcement can be formed on the weld surface by adding wire or powder to reduce the effect of surface thinning. Scholars focusing on the softening of the heat-affected zone have found that the hardness and tensile strength of welded joints increase to a certain extent as the heat input of friction stir welding decreases. The softening phenomenon of the heat-affected zone is also alleviated [14,15]. To reduce the effect of the heat input of the tool, water cooling is currently used instead of room-temperature cooling [16,17,18], or underwater friction stir welding is used [19,20,21] to decrease heat input and improve mechanical properties.

To solve problems on the surface thinning and the heat-affected zone, this study proposes friction stir welding with cover sheet (CFSW). Butt welding was performed for 2-mm-thick aluminum alloy sheets. Before welding, a 0.6-mm-thick aluminum alloy sheet was covered on the welded sheet. The idea of this design may bring the following potential advantages: (1) The cover sheet can supplement the thinning caused by the rotation of the tool shoulder to form reinforcement on the surface of the weld seam similar to gas metal-arc welding, thereby enhancing the strength of the weld area. When the joint shape is strictly required, reinforcement can be eliminated by mechanical processing to ensure a smooth surface. (2) The overlap defect in the welding process can be eliminated by mechanical processing without affecting the welded sheet metal. (3) The downward pressure of the shoulder on the workpiece is replaced by the cover sheet. The relatively low heat transfer efficiency between two sheets reduces the influence of the cyclic heat of the shoulder on the lower butt sheets and the area of the heat-affected zone. The effects of welding parameters on weld forming and mechanical properties were studied, and the stress distribution of the joint was analyzed.

## 2. Materials and Experiments

**Figure 1:**
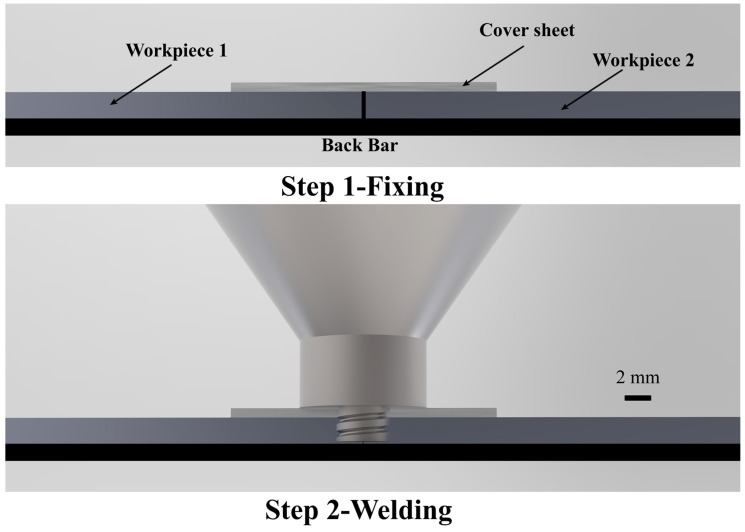
Sketch map of friction stir welding with cover sheet (CFSW).

The welding material was 2-mm-thick 5754H111 aluminum alloy. The cover sheet was 0.6 mm thick. The chemical composition of welded sheets was measured by a scanning electron microscope (SEM) equipped with energy dispersive X-ray spectroscopy (EDS), and mechanical properties were tested by a universal testing machine. The composition and mechanical properties of sheets are shown in Table 1 and Table 2. The sketch map of CFSW is shown in Figure 1. First, 2-mm-thick aluminum alloys were butted with the added 0.6-mm-thick cover sheet. Then, the welding was conducted along the butt gap. The diameter of the tool shoulder was 14 mm. A right-handed triple-inclined cylindrical threaded tool pin was adopted. The diameter of the tool pin was 3 mm. Different plunge depths were used to investigate its influence on strength in the experiments, so the tool pin length depended on the thickness of the cover sheet and the plunge depth. In the experiment, the inclination angle of the tool remained unchanged. Three parameters, namely, welding speed, rotational speed, and plunge depth, were changed, as shown in Table 3. 

**Figure 2:**
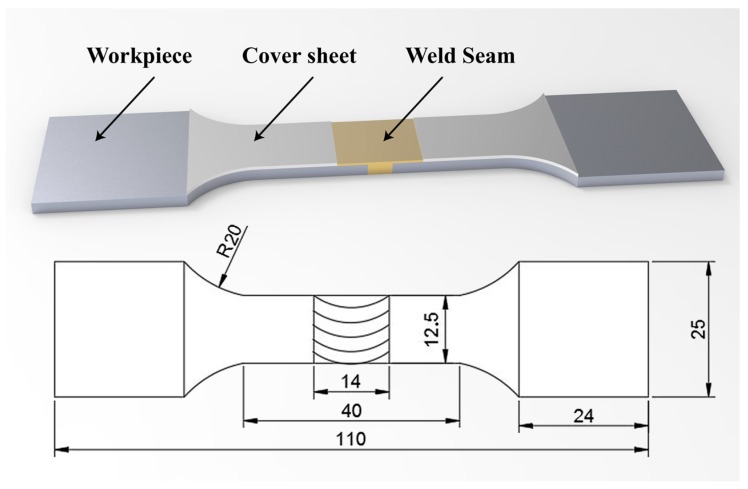
Dimensions of the tensile specimens.

After welding, tensile testing was carried out on the joint. The cover sheet was not machined. The tensile test was only for the lower butt sheet and the cover sheet was not restrained. The size of the tensile testing specimen is shown in Figure 2. The tensile speed was 2 mm/min and the average strength of three specimens was used as the strength value. All metallographic specimens were ground and polished (sand paper 120–2000, granularity of polishing agent 0.5 μm). Then, Keller’s reagent (2 mL of HF, 3 mL of HCl, 5 mL of HNO_3_, and 190 mL of H_2_O) was used to induce metallographic-phase apparent corrosion. The microhardness was measured in 0.5-mm intervals on the cross section of the welding specimen under 50 gf load and 10 s retention time.

Finite element software (ABAQUS) was used to simulate stress distribution in the tensile process for the 2 mm-thick conventional friction stir welding and tensile standard joint with added 0.6 mm cover. Subsidence in its tool shoulder was fixed at 0.2 mm. The weld zone was 4 mm wide, which was slightly more than the diameter of the tool pin. The rest of the cover sheet was not connected to the lower sheets. One end of the model was completely fixed, whereas the other end was shifted outside using an extended axis to simulate the tensile process (5 mm). An entity reduction integral unit (C3D8R) was used for calculation. A total of 35,836 units were observed in the finite element model.

## 3. Results and Discussion

### 3.1. Effects of Welding Parameters

#### 3.1.1. Cover Sheet

**Figure 3:**
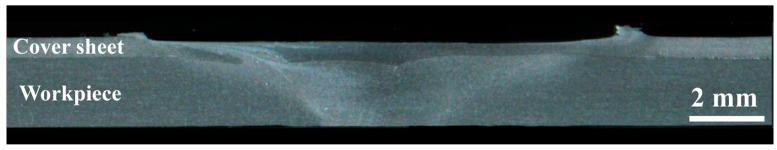
Cross section of the CFSW joint.

The cover sheet was selected on the basis of its material and thickness. A sheet with the same brand as the lower butt sheet and a dissimilar metal was selected. Although the cover sheet and the lower butt sheet had the same brand, the rolling process and microstructure were different. The thickness of the cover sheet depended on its weldability, the thickness of the lower butt material, and the stress distribution after welding. At the beginning of the experiment, 0.1–0.7-mm-thick aluminum alloy sheets were used as cover sheets. The insufficient rigidity of aluminum alloy will deform a sheet with a thickness less than 0.5 mm used as the cover sheet, which brings difficulty in forming a connection. Therefore, 0.6-mm-thick 5754 aluminum alloy was used as the cover sheet for welding. The cross section of a typical joint in CFSW is shown in Figure 3. After welding, the cover sheet was jointed with the lower butt sheet. The action of the weld zone showed a T-shaped distribution. As shown in the cross section, the joint only thinned in the cover sheet. This avoided the influence on the lower butt sheet.

#### 3.1.2. Plunge Depth

**Figure 4:**
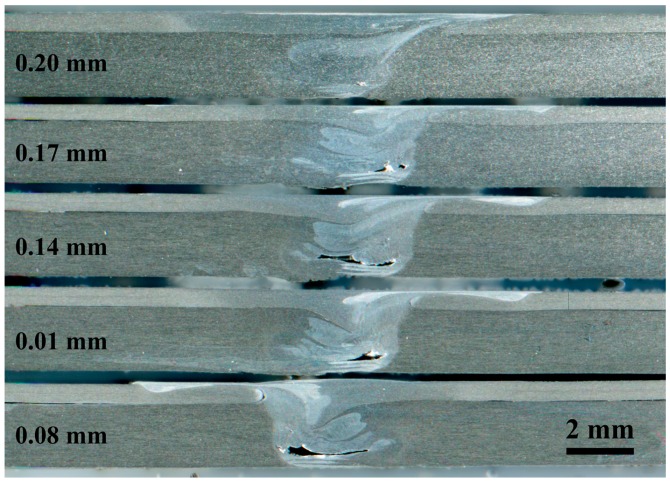
Cross section of the weld seam under different plunge depths.

The plunge depth of the tool shoulder is the most important factor affecting CFSW. The excessive plunge depth of traditional friction stir welding leads to various problems, such as serious thinning in the weld seam and excessive flash. After the cover sheet was added, the above-mentioned defects only occurred in the cover sheet and were removed by later mechanical processing. Welding under the plunge depths of 0.04, 0.08, 0.12, 0.16, and 0.20 mm was conducted. The results are shown in Figure 4. Insufficient plunge depth led to insufficient metal flow in the joint, voids, tunnels, and other defects in the bottom, and even surface grooves in serious cases. With the increase in the plunge depth, the hole defects at the bottom of the joint decreased gradually until they disappeared. In traditional friction stir welding, the tool shoulder directly acts on the sheet, thereby providing pressure and heat. After adding the cover sheet, the lower butt sheet had sufficient pressure and the cover sheet and the lower butt sheet were closely coupled to ensure high plunge depth.

#### 3.1.3. Heat Input

**Figure 5:**
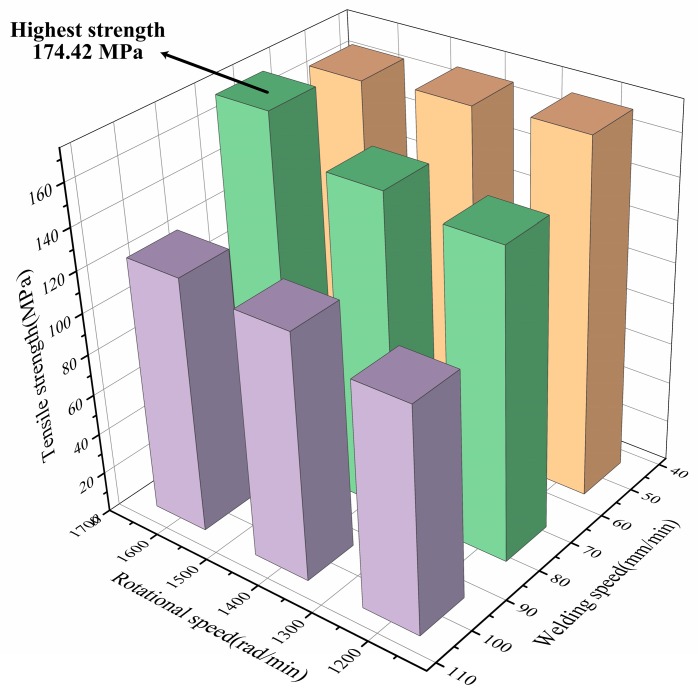
Relationship between welding speed, rotational speed, and the tensile properties of the joint.

Much of the heat of friction stir welding is produced from the shoulder friction. In this study, much of the heat produced by the shoulder was absorbed by the cover sheet. The heat required by the lower butt sheet for the joint was increased in the proportion from the stirring tool pin. The rotational and welding speeds determine the heat input in the welding process. At the beginning of the experiment, welding parameters were prescreened. The welding strength was difficult to guarantee, even if the welding speed was decreased to ensure heat input at low rotational speed. After the rotational speed reached 1200 r/min, the weldability improved accordingly. The mechanical properties were tested at the rotational speeds of 1200, 1400, and 1600 r/min and the welding speeds of 50, 75, and 100 mm/min. The results are shown in Figure 5. When the rotational speeds were 1200 and 1400 r/min, tensile strength increased with the decrease in welding speed. The joint strength was the highest when the rotational and welding speeds were 1600 r/min and 75 mm/min, respectively. The ultimate tensile strength of CFSW was 174.42 MPa, which is 94.11% of that of the base material. 

**Figure 6:**
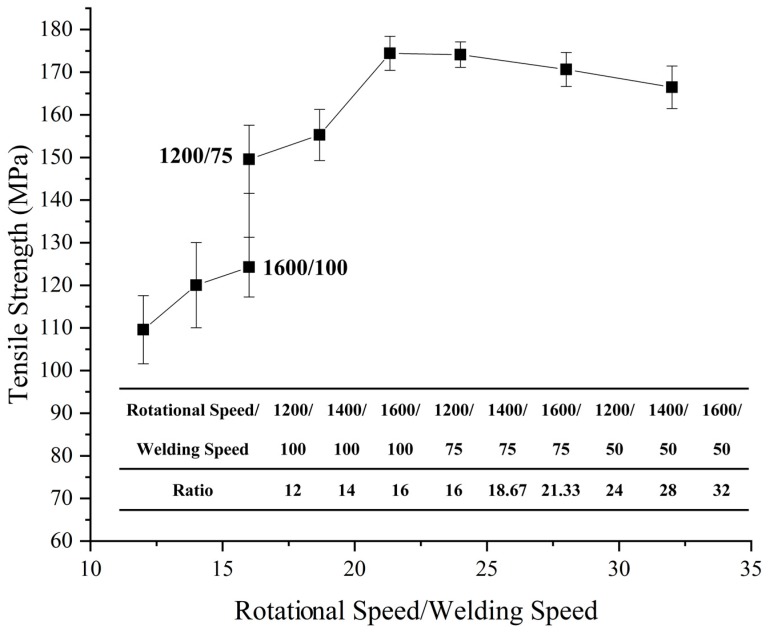
Relationship between welding line energy and mechanical properties.

Weld heat input is an important factor affecting the metallurgical and mechanical properties of the metal welding joint in the welding process. In friction stir welding, it is difficult to accurately calculate the energy from stirring and friction. Thus, the energy per unit length of weld can be approximately measured by the ratio of the rotational speed of the tool to the welding speed. The relationship between approximate linear energy and tensile strength under different welding parameters was analyzed. The results are shown in Figure 6. The welding strength first increased and then decreased with heat input. When the ratio was increased from 12 to 18.76, the welding strength increased rapidly and reached a peak at the ratio of 21.33. When heat input increased further, the welding strength slowly decreased. Therefore, the sheet was considerably sensitive to heat input because the cover sheet absorbed the heat produced by the tool shoulder in the welding process. As a result, the initial strength rapidly rose with the increase in heat. When the heat input reached the threshold of effective connection, the trend of strength stabilized. 

**Figure 7:**
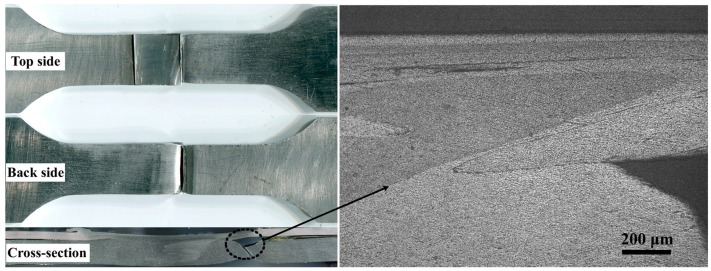
Morphology of the fracture of joints.

**Figure 8:**
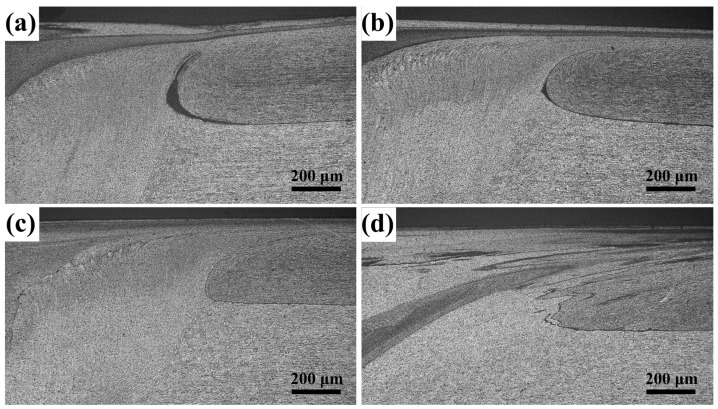
Morphology of the forward side of the edge of the weld seam under different heat input conditions, (**a**–**d**) refer to the ratio of rotational/welding speeds of 14, 16, 18.67, and 21.33.

In the tensile test, two fracture positions were observed in the joint. When the strength was low, fracture occurred in the middle of the weld seam. Lack of fusion was found in the fracture. When the welding strength was high, the fracture position of the joint was located outside the weld nugget, specifically on the forward side of the joint. A typical fracture joint is shown in Figure 7. In lap joints, hook and interlock occur in the interface between sheets as the tool rotates and moves. The starting position of the fracture was located at the edge of the hook region. The forward side under different heat input conditions is shown in Figure 8. If heat input was small, then the formation of an effective integration of the front edge of the upper and lower sheets was difficult. With the increase in heat input, the edge flow of the forward side intensified. The effective interlock between the upper and lower sheets was also gradually formed through the stirring. 

### 3.2. Microhardness

**Figure 9:**
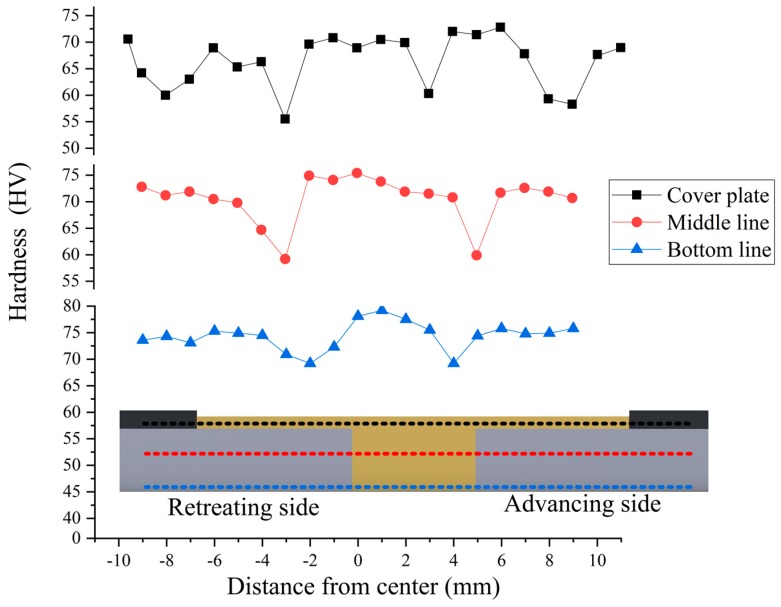
Microhardness of the joint.

The hardness of the lower surface of the cover sheet, the middle layer, and the lower surface of the lower butt sheet was tested. The results are shown in Figure 9. The hardness of the middle and the lower surface of the lower butt sheet was similar to that of conventional friction stir welding. In particular, the hardness showed a W-shaped distribution. The hardness decreased from base metal to heat-affected zone (HAZ) and reached the lowest at the junction of HAZ/ thermo-mechanically affected zone(HMAZ). Under the action of thermal cycling heat of the tool, overaging occurred in this area. As a result, the hardness decreased. The hardness gradually increased as it passed through heat-engine-affected and welding nugget zones. The maximum was slightly higher than that of the base metal. When entering the other side of the weld, the hardness trend of the front side was roughly repeated. Specifically, the hardness decreased first and then rose, and the minimum hardness values of the two sides were nearly the same. The heat input on the lower surface of the sheet was small, and the heat conduction of the cushion was fast. Consequently, the hardness of the heat-affected zone on the middle layer was lower than that on the bottom layer.

The hardness distribution on the lower surface of the cover sheet was different from those on the middle and lower surface of the lower butt sheet. Four zones had decreased hardness, and they were located at the outer side of the tool shoulder acting zone and the outer side of the tool pin acting zone. In the outer side of the shoulder acting zone, the cover sheet was affected by the heat cycle. As a result, a heat-affected zone was formed, which decreased the hardness. After entering the direct acting zone of the tool shoulder, the hardness of the cover sheet increased due to the stirring action. When reaching the outer side of the tool pin, the metal between the cover sheet and the lower sheet flowed in an axial direction due to the effect of the tool pin thread. This condition changed the metal distribution in this area and caused the softening of this site.

An advantage of CSFW is that the width of the heat-affected zone can be decreased. In traditional friction stir welding, the tool shoulder provides the most heat input. As a result, the width of the heat-affected zone is more than the outer diameter of the shoulder. When CSFW was adopted, the tool shoulder pressed on the cover sheet. Therefore, the heat only entered the lower butt sheet through heat conduction at the interface between the cover sheet and the lower butt sheet. The width of one side was only 2 mm, which was smaller than the outer diameter of the shoulder. This reduced the influence of excessive heat input on the welding quality.

### 3.3. Stress Analysis

**Figure 10:**
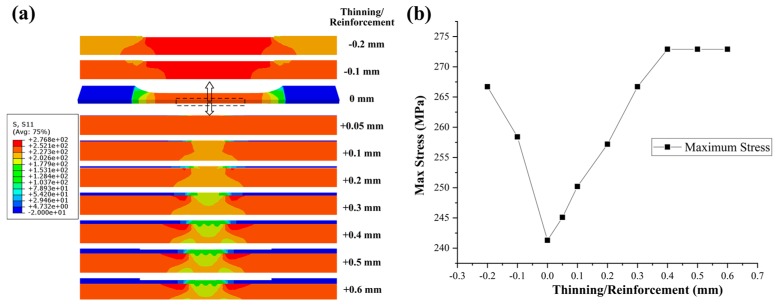
Comparative stress analysis under different thinning amounts and reinforcements: (**a**) comparison of stress distribution map and (**b**) comparison of maximum stress.

The tool shoulder of traditional friction stir welding leads to inevitable surface thinning after welding. In general, the thinning amount should not be greater than 10% of the sheet thickness. After the cover sheet was added, thinning of the welded sheet was avoided. Reinforcement on the welded sheet after welding is sometimes unnecessary, so it can be retained or removed by mechanical processing. Comparative stress analysis of joints with different thinning amounts and reinforcement conditions was conducted by numerical simulation. Notably, the differences in the performance of materials in different areas were ignored in the simulation. Only the stress distribution caused by the structural factor was considered. A displacement of 5 mm was added to the joint, and the results are shown in Figure 10. When no cover sheet was added, two thinning conditions (i.e., 0.1 and 0.2 mm) were selected for analysis. In the case of CSFW, the thinning condition of 0.2 mm produced in the cover sheet was selected to perform different milling operations. When the weld was thinned, the stress concentration was formed in the thinning area in the tensile test. The maximum stress increased with the increase of thinning. After the cover sheet was added, the stress concentration shifted from the weld area to outside of the weld due to reinforcement formed on the weld. However, with the increase in reinforcement, the stress concentration at the edge and corner of the weld increased. The concentration area was also enlarged. The maximum stress increased with the increase in reinforcement. However, when reinforcement reached 0.4 mm, the maximum stress did not change. When reinforcement was less than 0.2 mm, the maximum stress was less than that by traditional friction stir welding in the thinning condition of 0.1 mm. 

## 4. Conclusions

To solve thinning produced by the shoulder and reduce the heat-affected zone, this study proposes the CSFW and analyzes its microstructure and mechanical properties. The following conclusions are made:

(1) CSFW could improve surface shaping of the weld and overcome the subsidence of the weld surface from downward press of the shoulder, negative effects from the overlap, and other problems caused by the metal loss.
(2) The cover sheet absorbed the heat from the shoulder. Thus, CFSW needs high heat input to realize the welding shaping. With the increase in heat input, the stirring action on the forward side increased, thereby forming mechanical occlusion and improving joint strength. In the tensile test of 5754 aluminum alloy, the fracture site of the joint was located at the edge of the forward side. The maximum tensile strength was 94% of that of the base metal.
(3) The hardness distribution on the cross section of the joint cover sheet and the lower sheet showed a W-shaped distribution. The width of the heat-affected zone was 2 mm. The cover sheet absorbed the heat produced by the shoulder. As a result, the width of the heat-affected zone of the lower butt sheet was smaller than the diameter of the shoulder.
(4) After the cover sheet was added, stress concentration shifted from the thinning site by traditional friction stir welding to the outer side of the weld. With the increase in reinforcement, the stress concentration zone enlarged.

## Figures and Tables

**Table 1:** Chemical composition of AA5754 H111 alloy (%).

Mg	Mn	Si	Fe	Cr	Zn	Ti	Cu	Al
2.78	0.35	0.17	0.28	0.12	0.09	0.07	0.06	Balance

**Table 2:** Mechanical properties of AA 5754 H111 alloy.

Yield Strength /MPa	Tensile Strength /MPa	Elongation	Elastic Modulus /GPa	Poisson’s Ratio
80 MPa	177 MPa	14%	67	0.33

**Table 3:** Friction stir welding with cover sheet (CFSW) process parameters during experiments.

Experiments	Welding Speed (mm/min)	Rotational Speed (RPM)	Plunge Depth (mm)	Tilt Angle (°)
1	100	1200	0–0.2	1
2	100	1400		
3	100	1600		
4	75	1200		
5	75	1400		
6	75	1600		
7	50	1200		
8	50	1400		
9	50	1600		
